# The bioinformatics aspects of gene screening of HT-29, human colon cell line treated with caffeic acid 

**Published:** 2019

**Authors:** Majid Rezaei-Tavirani, Mostafa Rezaei Tavirani, Mona Zamanian Azodi

**Affiliations:** 1 *Proteomics Research Center, Faculty of Paramedical Sciences, Shahid Beheshti University of Medical Sciences, Tehran, Iran*; 2 *Proteomics Research Center, Shahid Beheshti University of Medical Sciences, Tehran, Iran *

**Keywords:** Coffee consumption, Colon cancer, Transcriptome, Protein-protein interaction network analysis, Biological process analysis

## Abstract

**Aim:**

To better understand the anticancer properties of coffee, bioinformatics analysis of gene expression profile of HT-29, human colon cell line in the exposure of caffeic acid (CA) is proposed in this study.

**Background:**

Coffee as a popular beverage has been shown to be a potential health promoter as well as being effective on different kinds of diseases including cancer.

**Methods:**

The differentially expressed genes (DEGs) across the comparison of groups of samples including none-treated HT-29 and HT-29 tread with CA were applied for the protein-protein interaction mapping. Cytoscape v.3.7.1 constructed a network and analyzed the topological features of the most noteworthy DEGs.

**Results:**

The genes of CTSZ, AFF4, DHRS2, and HMGCS1 known as active agents in cancer progression, were identified as the central DEGs in the constructed PPI network in this study. Especially, HMGCS1 is the most central gene is also linked to one of the important colon cancer altered processes, cholesterol metabolism.

**Conclusion:**

Lowering the risk of colon cancer could be perhaps through nominated DEGs and therefore, regulation of serum cholesterol as well as protection against cancer development.

## Introduction

 Cancer of colon is one of the most common kinds of malignancies in the world with a report of one million new cases each year (1). The available potential therapy approaches for cancer is chemotherapy and chemoradiotherapy. Nevertheless, the application of these methods can have some side effects in human body as well, due to their toxicity properties on normal cells. Some of these problems are some organs dysfunction (2) and anemia (3), and symptoms including nausea, vomiting, anorexia, diarrhea, oral mucositis, and numbness that may occur and could influence patients’ daily life (4). In view of this fact, there is a need for finding a cure with less complications for cancer prevention and treatment (5). On the other hand, coffee has been shown to be a healthy beverage for human. It has many positive effects on human body including reducing the chance of different kinds of diseases such as diabetes type 2, cancer, dementia, depression, and cirrhosis (6-10). In addition, the anticarcinogenic properties of coffee in different types of cancers is also suggested by some studies. Oral cavity cancer (11), endometrial cancer (12) and colon cancer (13) are some of which, that are influenced by the application of coffee treatment. However, there is a contradictory notion about the exact effect of coffee on these cancers. Some claims that coffee could increase the chance of cancer grow while others imply on anticancer properties of coffee intake (14). The possible preventive outcome of this regular consuming food popular in western countries is due to its potential chemicals (15). To understand the mechanisms by which coffee plays protective role in different kinds of diseases, many molecular studies have been conducted (16, 17). The most promising high throughput studies such as genomics, proteomics, and metabolomics could provide more information in this sense (18-20). As mentioned, one of them is microarray gene expression profiling that has been used widely in the recent years as a suitable method for understanding disease molecular changes. Furthermore, the data produced by this type of analysis can be used for bioinformatics in terms of interactions and gene ontology evaluations (21). In fact, differentially expressed genes (DEGs) could produce more information related to their centrality properties in a interaction map (22) in addition to their expression alterations. Here, the worthy values of coffee consumption are explored on HT29 cell lines by the application of PPI network analysis and its corresponding algorithms. It is also aimed to introduce plausible biomarkers and underlying processes that play crucial role in coffee chemopreventive mechanisms. 

## Methods

The study conducted by Oleaga C and *et al.* identified and compared the expression difference between three groups of samples (HT29 colon cells treated with instant coffee and caffeic acid (CA) versus control) from human model. The research was entitled as “Comparison of gene expression profiles of HT29 cells treated with instant caffeinated coffee or CA versus control” and also published as “Coffee polyphenols change the expression of STAT5B and ATF-2 modifying cyclin D1 levels in cancer cells” in 2012 (19). In our approach, the comparison is between the samples of control and the samples treated with none toxic dosage of CA, 1.68 µ/ml for 24h. This low concentration of CA treatment is equal to one cup of coffee. All these information is provided from GEO, Gene Expression Omnibus, https://www.ncbi.nlm.nih.gov/geo/. Expression profile, GSE35382 was based on platform, GPL570, [HG-U133_Plus_2] Affymetrix Human Genome U133 Plus 2.0 Array. The differentially expressed genes from this comparison was obtained from the online analyzer, GEO2R. This tool is available in GEO and it orders genes based on their significance in expression. This analysis is based on R programming language using (GEOquery and limma R packages). Prior to comparing the groups of interest, the cross-comparison between groups via box plotting was carried out to realize whether they are qualified for the evaluation. The median-centered data show that groups are fit for this purpose and the analysis could be continued. One of the assigned criteria for the determining the DEGs is the fold change (FC). Here, the FC is set to the cut off = 1.5 and the p.value ≤ 0.05. For the network analysis, at first the genes without symbols were omitted and then those with names and significantly expressed across the analysis were chosen for the network construction. The network application for this study was Cytoscape v. 3.7.1 and the corresponding plug-ins installed in this software. String DB applied in Cytoscape was the source for the network query (23, 24). The pattern of differentially expressed genes across the comparison between groups of samples were then depicted by one of the Cytoscape software designated for enrichment analysis of the query elements including genes, miRNAs, and proteins. The so called plug-in is CluePedia that by assigning expression profile file from GEO Database, visualize the expression pattern of the analyzing genes (25). The next step is to perform enrichment analysis that is generally consists of biological process (BP), molecular function (MF), cellular component (CC), and KEGG pathways. Here, BP investigation is considered for the designated elements of the study with the following statistical criteria: GO term min number of genes=1 and min gene percentage in term=1. Bonfferoni step down is the correction method for p-value. Two-sided hypergeomtric test is the enrichment/depletion. 

## Results

**Figure 1:**
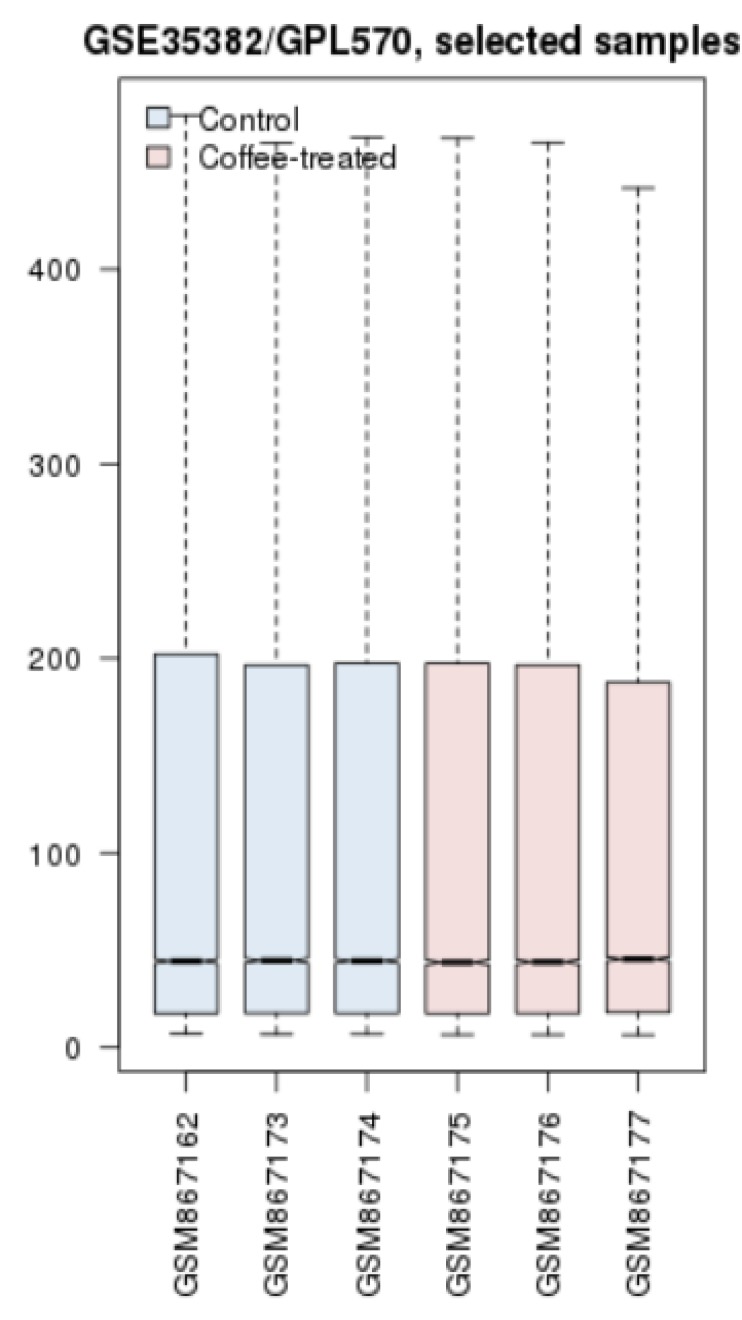
Cross comparison with the use of box plot method for control (blue) and coffee treated groups (pink). The line crossing the boxes indicates that the gene expression pattern for the samples is either very low or very high.

To evaluate if the samples are comparable in terms of expression, cross comparison method was applied as box plotting in figure 1 for control and coffee exposed samples. The analysis from the box plotting implies that the samples are all median-centered and proper for further analysis in terms of identifying the differentially expressed genes. Genes with altered statistically significant expression (p-value ≤ 0.05) and FC ≥ 1.5 are gathered in table 1.

**Table 1:** The list of significantly differential expressed genes and their corresponding statistical properties obtained by GEO2R analysis, p-value ≤ 0.05, FC ≥ 1.5

**Row**	**Gene. Symbol**	**FC**	**P-value**	**Regulation Type**
1	HINT3	2.6	0.002	Up
2	SIAE	2.4	0.0003	Up
3	FGD4	1.5	0.003	Down
4	S100A2	1.5	0.0004	Down
5	CTSZ	1.5	0.006	Down
6	SLC4A4	1.5	0.00006	Down
7	AGR3	1.7	0.0007	Down
8	PURB	1.7	0.009	Down
9	HMGCS1	1.7	0.010	Down
10	AFF4	1.5	0.014	Down
11	ERGIC1	1.5	0.014	Down
12	LGALS2	1.5	0.016	Down
13	C5orf24	1.6	0.03	Up
14	DHRS2	1.6	0.03	Up

**Figure 2:**
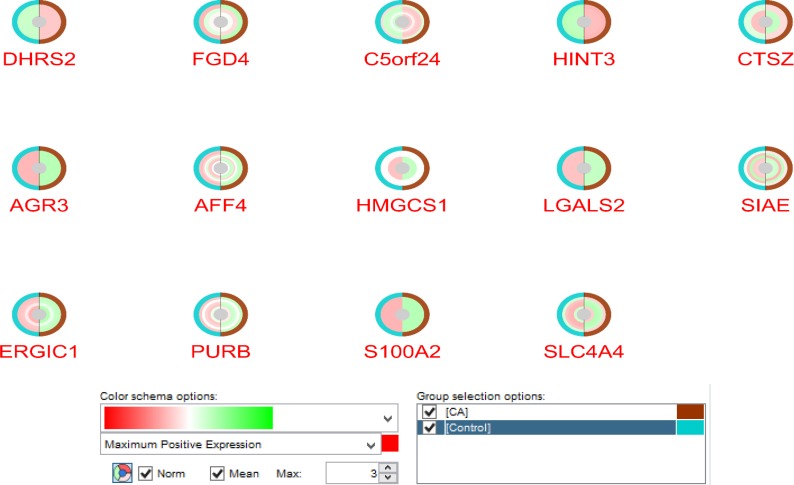
The normalized and mean expression pattern for the 14 genes in two compared groups via CluePedia software. The control groups are shown by blue color and the coffee extract treated subjects are in brown color. In addition, the color alteration from red to green indicate the expression maximum positive expression changes to negative expression. White circles around genes express null expression values

**Table 2:** The list of DEGs with their corresponding centrality values in the constructed network. BC stands for betweenness centrality

R	Gene	Description	Degree	BC
1	HINT3	histidine triad nucleotide binding protein 3	1	0
2	SIAE	sialic acid acetylesterase	1	0
3	FGD4	FYVE, RhoGEF and PH domain containing 4	2	0
4	S100A2	S100 calcium binding protein A2	1	0
5	CTSZ*	cathepsin Z	9	0.14
6	SLC4A4	solute carrier family 4, sodium bicarbonate cotransporter, member 4	1	0
7	AGR3	anterior gradient 3 homolog (Xenopus laevis)	0	0
8	PURB*	purine-rich element binding protein B	2	0.01
9	HMGCS1*	3-hydroxy-3-methylglutaryl-CoA synthase 1 (soluble)	25	0.11
10	AFF4*	AF4/FMR2 family, member 4	12	0
11	ERGIC1	endoplasmic reticulum-golgi intermediate compartment (ERGIC) 1	2	0
12	LGALS2	lectin, galactoside-binding, soluble, 2	0	0
13	C5orf24	Chromosome 5 open reading frame 24	1	0
14	DHRS2*	dehydrogenase/reductase (SDR family) member 2	13	0

**Figure 3:**
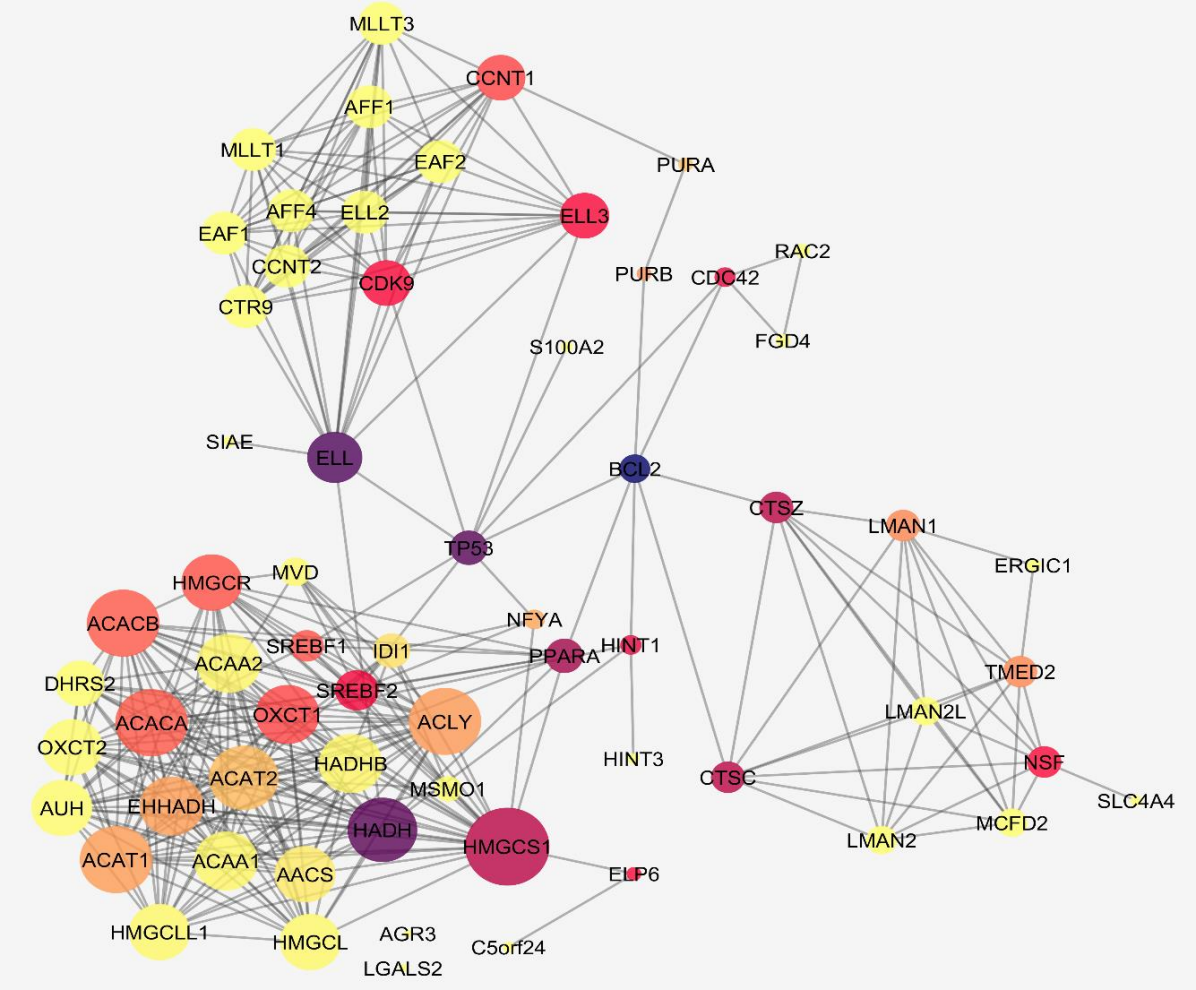
A network of interacting DEGs with the addition of 50 neighbors. The color spectrum and the node size changes indicate the betweenness value and degree alterations, respectively. From yellow to blue, the scheme shows the higher values of BC likewise the bigger the nodes the higher the degree amount

**Figure 4:**
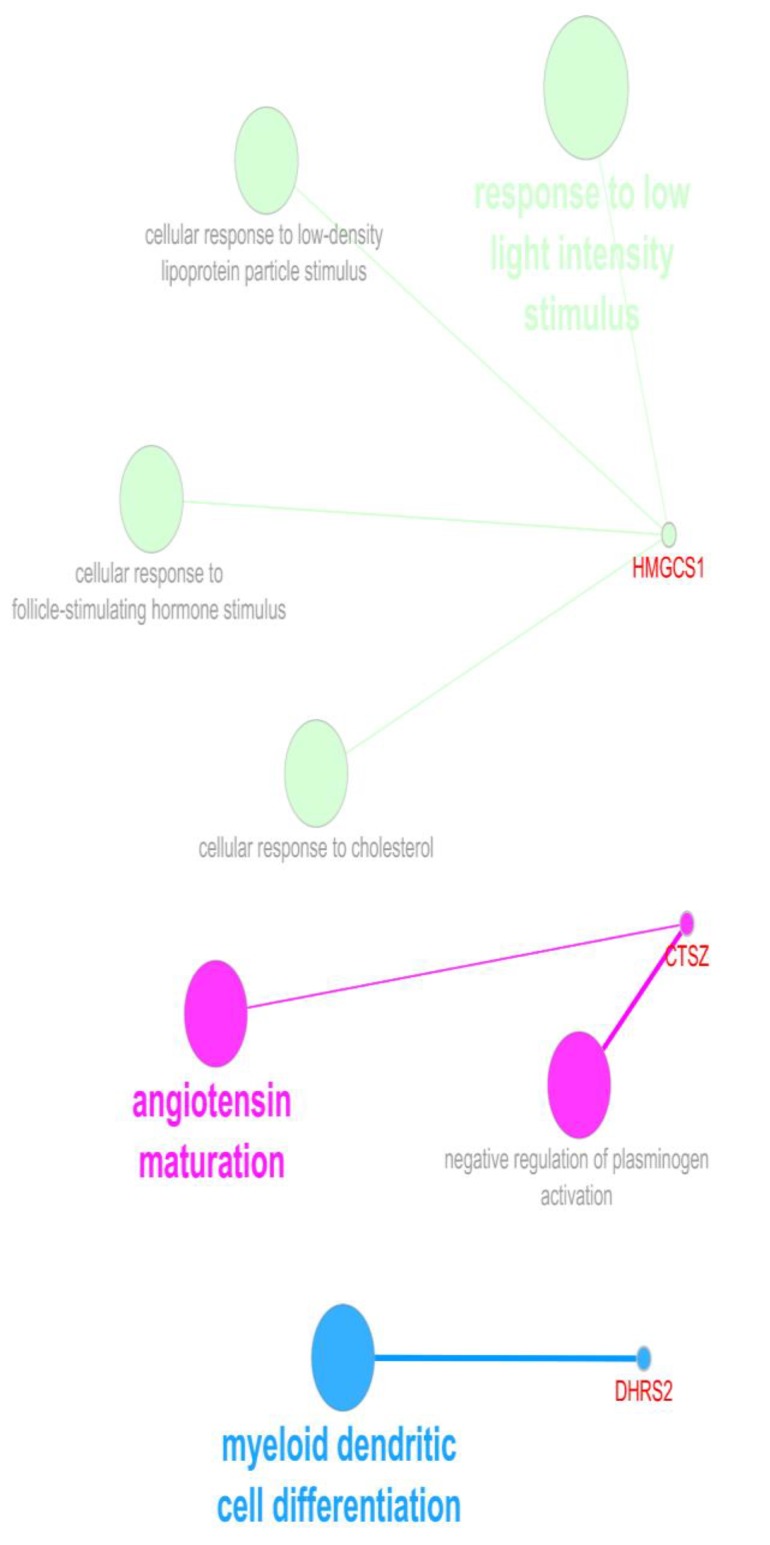
The BPs of the queried four genes resulted in three identified groups of terms for each three genes. It should be noted that AFF4 was not recognized in this query. Gene ontology tree interval

In table 1, the most genes are down-regulated by the treatment of coffee extract in HT-29 cells. The eight first genes are among the top 250 significant ones. The first two up-regulated and the last six ones are down-regulated. The other six ones were from the rest of differentially expressed genes.

Protein interaction network can be analyzed in regards to centrality properties. DEGs that are also central nodes could have more prominent role in the disease pathology as well as being valuable targets for treatment goals. 

Examining expression pattern of the 14 elements in GSE35382 study that could provide visualization of the changes across groups as depicted in figure 2.

In figure 2, all of these genes possess significant expression difference in at least one spot. These changes are depicted as normalized and mean values of samples for each spots. All spots of DHRS2, HINT3, AGR3, LGALS2, and S100A2 are with significant differential changes across comparing two sets of groups. 

In figure 3, a centrality analysis related to degree for 14 queried genes with their 50 neighbor genes is shown. A constructed network is consisted of 64 nodes and 336 interactions. 

To identify the most important DEGs, a centrality analysis through network construction provide more information. These genes are focused for degree and betweenness centrality analysis as tabulated in table 2. Most of these genes do not reach high values of centrality. Only four genes including CTSZ, HMGCS1, AFF4, and DHRS2 have the highest amounts. HMGCS1 shows to be potential in this analysis.

Figure 1. Cross comparison with the use of box plot method for control (blue) and coffee treated groups (pink). The line crossing the boxes indicates that the gene expression pattern for the samples is either very low or very high. 

In figure 4, three genes from four query ones were obtained from the analysis and the terms associated with these genes are as follow: response to low light intensity stimulus, angiotensin maturation, and myeloid dendritic cell differentiation that HMGCS1, CTSZ, and DHRS2 are linked to these BPs, respectively.

In the next step, the biological processes (BPs) of the four DEGs with the highest centrality properties including CTSZ, HMGCS1, AFF4, and DHRS2 were queried via ClueGO+ CluePedia plug-ins in figure 4.

## Discussion

The second rate for cancer related death in the world is for colon cancer (26). Diet has a strong relationship with the incident of this type of cancer. Consumption of food with high amount of fat and low fibers could increase the chance of colorectal cancer (27). It is suggested that plant-based intake can reduce its incidence (28). One of which is coffee with significant positive preventive effect on colon cancer development (29). In addition, molecular studies could be helpful to decipher the coffee significance as an anticancer agent. Therefore, bioinformatics analysis of gene expression profile of colon cancer in exposure to coffee extract has been conducted to better understand its chemo-preventive mechanism. The applied bioinformatics method is PPI network evaluation of the cell line of HT-29 from the database of GEO. The GEO database offers an online tool called GEO2R that does the comparison analysis of the designated groups of samples of interest. In our study, across comparison between control samples and CA treated ones showed some genes with differential expression. However, at first the groups were compared via a statistical approach, box plot analysis, to understand whether they are eligible for the study. In figure 1, the analysis is depicted and it is approved that the groups are valid for continuing the analysis. The cross-comparison identified 14 altered expressed genes as listed in table 1. Among these 14 genes, there are some central ones that play more influential role in coffee exposure. In other words, genes with topological features may propose as better targets for therapy approaches. As we explore the DEGs, it can be inferred from table 1 that most of these agents are down regulated in the presence of CA. Moreover, when look into their pattern of expression changes in figure 2, there are genes that are significant in all their spots while some have different ranges. Network Analyzer provided the centrality properties of the constructed network and showed key genes in the network strength in figure 3. Most of these key genes are from neighbor interacting genes with the DEGs. At a glance, it is clear that HMGCS1 is one of the highlighted DEGs that express high values of centrality. To focus more on the DEGs, the centrality values of all 14 genes were then examined in table 2 with the relating two parameters of degree and betweenness centrality. The exploration identified that only four genes among these nodes are valuable in network stability and the rest declare not much of interacting elements in the provided network. Some of these four agents are important for both criteria and some are either for degree or for BC. HMGCS1 as mentioned earlier in the first look in to network analysis is among the essentials of top ranked central nodes (hub-bottleneck). 

The other three genes namely, CTSZ, AFF4, and DHRS2 as well have some major roles in the network. CTSZ displays high betweenness amount that could be considered as a bottleneck. The last two ones, AFF4 and DHRS2 have moderate degree values that may also be prominent for the network interactions. Further analysis of the concentrating significant differentially expressed genes offers that there are some chief biological processes related to them as illustrated in figure 4. The CA can alter these processes functions as treated with HT-29. To see what impact these genes could have in cancer in response to coffee treatment, their linkage to cancer is then reviewed by literature as well. The whole analysis offers that HMGCS1 is identified as a statistically significant down-regulated gene in one spot and one with no differential expression in CA treatment. 3- hydroxy-3-methylglutaryl-CoA synthase 1 (soluble), HMGCS1 is located on chromosome 5 in which its changes is linked to cancer progression (30). This transferase enzyme is corresponding to cholesterol metabolism, cellular response to cholesterol and biosynthesis process that has a complex contribution in colorectal cancer. HMGCS1 encodes Hmg-CoA synthase 1 that contributes to the first reaction of cholesterol synthesis by catalyzing acetyl CoA and acetoacetyl-CoA hydration to 3-hydroxy-3-methylglutaryl CoA (Hmg-CoA) (31). On the other hand, it has been reported that lower cholesterol levels of serum could be an indication of this kind of cancer development (30, 32). Moreover, coffee consumption elevates the cholesterol levels in the serum (33, 34). So, one of the mechanisms by which CA impact on metabolism of colon cancer could be through expression alterations of HMGCS1. In other words, this finding could help justifying the intricate associations of colorectal cancer and cholesterol metabolisms. Cathepsin Z the next gene, is an enzyme likewise the previous gene and it acts as protease which is down-regulated for two spots in CA treated sample. These spots are one with statistically down regulation in the CA treated while the other one is with no statistically differential expression in treated sample. In addition, it has a great betweenness value; however, not a good choice as hub. Therefore, it is called bottleneck of the constructed network. The up-regulation of this enzyme family has a fundamental association in promoting cancer development (tumorigenesis and invasion) (35, 36). By the application of coffee, the expression levels of this gene expression changes dramatically to statistical significance of 1.5 lower. Likewise, DHRS2 as dehydrogenase/reductase (SDR family) member 2 is also an enzyme that contains two spots in this investigation and both are up-regulated considerably. Furthermore, this gene is with reasonable high connections in the constructed network in terms of degree values. The function of this gene in some type of cancers such as esophageal squamous is to inhibit tumor development. The low expression of this gene is demonstrated in this type of cancer (37). Coffee could regulate the levels of this gene in colon cancer by elevating the expressions. So, it appears that CA virtually effects on three prominent enzymes in colon cancer. AFF4 as AF4/FMR2 family, member 4 is another relatively central node with moderate degree levels; however, it could not be considered as a hub gene. This gene also has a slight different pattern of expression changes in spots of CA treated sample. In which, nine spots are available for AFF4 that seven of them are down-regulated and two up-regulated. Among them, only one that is down-regulated is statistically significant in differential expression. The previous studies showed that this gene is also related to tumor progression in some sorts of cancers including head and neck carcinoma and colon cancer (38, 39). Here, the expression level of this gene is regulated by CA treatment. Taken together, the alterations of DEGs in colon cancer may develop neoplastic behavior and coffee extract, on the other hand, could reduce these changes by interfering in inhibiting carcinogenesis. Especially, HMGCS1 as the most central gene among DEGs and with a distinct involvement in colon cancer purposed by previous findings (30), deserves further exclusive studies. 

To sum up, regulation of cholesterol metabolism and neoplastic developments could be parts of mechanisms in which coffee extract plays as a chemo-preventive agent in colon cancer. The candidates of constructed PPI network are involved in the mentioned processes, and therefore can be targets for facilitating colon cancer screening and therapy.

## Conflict of interests

The authors declare that they have no conflict of interest.
